# Protective Effects of Frankincense Oil on Wound Healing: Downregulating Caspase-3 Expression to Facilitate the Transition from the Inflammatory to Proliferative Phase

**DOI:** 10.3390/ph18030407

**Published:** 2025-03-13

**Authors:** Krishnaraju Venkatesan, Durgaramani Sivadasan, Moufida Abderrahmen Al Weslati, Mohammed Gayasuddin Mouid, Manoj Goyal, Monika Bansal, Mohamed EL-Dosoky Mohamed Salama, Syed Azizullah Ghori, Fazil Ahmad

**Affiliations:** 1 Department of Pharmacology, College of Pharmacy, King Khalid University, Abha 62521, Saudi Arabia; 2 Department of Pharmaceutics, College of Pharmacy, Jazan University, Jazan 45142, Saudi Arabia; dsivadasa@jazanu.edu.sa; 3 Department of Respiratory Care and the Department of Basic Sciences, College of Applied Medical Sciences in Al Jubail, Imam Abdulrahman Bin Faisal University, Al Jubail 38516, Saudi Arabia; maalweslati@iau.edu.sa; 4 Department of Basic Sciences, College of Applied Medical Sciences, King Saud bin Abdulaziz University for Health Sciences, Al-Ahsa 31982, Saudi Arabia; gayasuddin@ksau-hs.edu.sa; 5 King Abdullah International Medical Research Center, Al-Ahsa 31982, Saudi Arabia; 6 Department of Anesthesia Technology, College of Applied Medical Sciences in Jubail, Imam Abdulrahman Bin Faisal University, Jubail 35816, Saudi Arabia; mggoyal@iau.edu.sa (M.G.); fmahmad@iau.edu.sa (F.A.); 7 Department of Neuroscience Technology, College of Applied Medical Sciences in Jubail, Imam Abdulrahman Bin Faisal University, Jubail 35816, Saudi Arabia; mbbanasl@iau.edu.sa (M.B.); mesalama@iau.edu.sa (M.E.-D.M.S.); 8 Department of Pharmacy Practice, College of Clinical Pharmacy, Imam Abdulrahman Bin Faisal University, Dammam 31441, Saudi Arabia; agsayed@iau.edu.sa

**Keywords:** frankincense oil, wound healing, *Boswellia species*, oxidative damage, inflammatory cytokines, caspase-3 expression, immune cell markers

## Abstract

**Background/Objectives:** Wound healing is a complex process involving inflammation, oxidative stress, immune modulation, and tissue regeneration. Frankincense essential oil (FEO), derived from *Boswellia* species, is known for its anti-inflammatory, antioxidant, and therapeutic properties. This study investigates the protective effects of FEO in an excision wound model in rats, focusing on oxidative stress reduction, inflammatory cytokine modulation, and caspase-3 regulation. **Methods:** The chemical composition of FEO was analyzed using gas chromatography-mass spectrometry (GC-MS). Rats with excision wounds were treated with FEO, and its efficacy was assessed using biochemical and histological analyses. Caspase-3 expression, IL-1β, TNF-α, and CD68 levels were measured, along with oxidative stress markers. Wound contraction, epithelialization and collagen synthesis were also evaluated. Immunohistochemical and histopathological assessments were performed to analyze inflammatory infiltration and tissue remodeling. **Results:** FEO, rich in *alpha-phellandrene* (10.52%) and *limonene* (7.31%), significantly downregulated caspase-3, reducing apoptosis in the wound environment. It also lowered IL-1β and TNF-α levels, confirming anti-inflammatory effects. Additionally, FEO modulated CD68 expression, shifting the wound environment from inflammatory to healing. The oil antioxidant activity reduced oxidative stress, limiting caspase-3-mediated apoptosis and enhancing cell survival. FEO treatment accelerated wound contraction, improved epithelialization, and increased collagen synthesis. Histological analysis revealed reduced inflammatory infiltration and enhanced tissue remodeling. **Conclusions:** FEO integrates anti-inflammatory, antioxidant, and anti-apoptotic mechanisms to promote wound healing and tissue repair. Its ability to modulate caspase-3, IL-1β, TNF-α, CD68, and oxidative stress markers along with its major constituents such as *alpha-phellandrene* and *limonene* highlights its potential as a natural therapeutic agent for wound management and regenerative medicine.

## 1. Introduction

Wounds are disruptions in the integrity of the skin or underlying tissues caused by physical, chemical, or biological factors [1]. The wound healing process is a multifaceted and dynamic biological response comprising interrelated stages: hemostasis, inflammation, proliferation, and remodeling [2]. Together, these stages work harmoniously to restore the skin protective barrier and repair the damaged tissues. Effective wound management prevents complications such as infections and promotes faster recovery [3].

Hemostasis is the initial stage where vascular constriction and clot formation occur to stop bleeding. This is followed by the inflammatory phase, characterized by immune cell infiltration to remove debris and prevent microbial infections [4]. The proliferation phase involves the formation of new blood vessels (angiogenesis), fibroblast migration, and the deposition of extracellular matrix. Finally, during the remodeling phase, collagen is reorganized, and the wound gains tensile strength over time [5].

FEO, derived from the resin of the *Boswellia* tree, has been historically used for its medicinal properties, including wound healing [6]. The oil contains bioactive compounds such as boswellic acids, which exhibit anti-inflammatory, antimicrobial, and antioxidant properties [7]. These attributes make it a valuable agent in promoting wound repair. FEO helps reduce inflammation in the wound area, creating an optimal environment for healing [8]. Its antimicrobial effects help prevent infections, while its antioxidant properties combat oxidative stress, which can delay healing [9].

When compared to synthetic drugs, FEO offers several advantages in wound healing. Firstly, it has a lower risk of side effects and is generally well-tolerated when used appropriately [10]. Unlike synthetic antimicrobial agents, which may contribute to antibiotic resistance, FEO natural antimicrobial properties offer an alternative with minimal resistance development. Moreover, its antioxidant and anti-inflammatory effects provide comprehensive benefits that synthetic drugs often achieve through multiple medications [11]. However, due to their standardized formulations and targeted mechanisms, synthetic drugs may offer faster action and more consistent results. Synthetic options also undergo rigorous testing, ensuring predictable efficacy and safety, whereas natural remedies like FEO require further studies for standardization and dosage optimization [12].

Studies suggest that FEO enhances the proliferation phase by stimulating fibroblast activity and encouraging granulation tissue formation [13]. Additionally, it aids in the remodeling phase by promoting collagen synthesis, improving the tensile strength of the wound, and minimizing scarring. The oil is often used in diluted forms for topical applications, such as ointments or balms, and is increasingly integrated into modern wound care products [14].

This study evaluates the role of FEO in wound healing. Multiple indicators related to wound recovery were assessed, including body weight, feed intake, wound closure %, histopathological score, inflammatory cytokines (IL-1β and TNF-α), and macrophage activity (CD68). Furthermore, it evaluates oxidative stress indicators like reactive oxygen species (ROS), superoxide dismutase (SOD), malondialdehyde (MDA), and glutathione (GSH), along with the re-epithelialization process.

## 2. Results

The results show that the groups wound healing times varied significantly. It took up to 19 days for the wounds in the reference and control groups to heal. On the other hand, FEO-treated wounds showed a markedly faster healing process, demonstrating the oil strong therapeutic effectiveness.

### 2.1. Chemical Characterisation of Essential Oil

**Figure 1:**
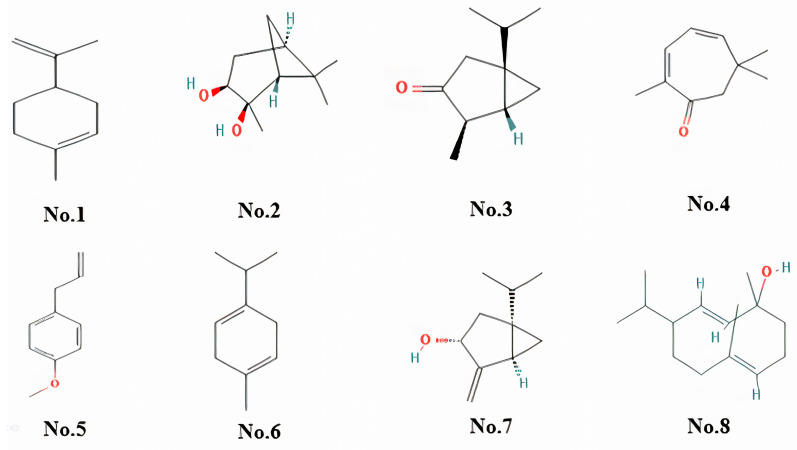
Structure of major compounds identified in an FEO- (**No.1**). Structure of Cyclohexene, 1-Methyl-4-(1-Methylethenyl), (**No.2**) structure of (1S,2S,3R,5S)(+)-Pinanediol, (**No.3**) Thujone, (**No.4**) 2,4-Cycloheptadien-1-One, 2,6,6-Trimethyl, (**No.5**) Estragole, (**No.6**) 1,4-Cyclohexadiene, 1-Methyl-4-(1-Methylethyl), (**No.7**) Bicyclo[3.1.0]hexan-3-ol, 4-methylene-1-(1-methylethyl)-, (1.alpha.,3.alpha.,5.alpha.), (**No.8**) (2E,4S,7E)-4-Isopropyl-1,7-dimethylcyclodeca-2,7-dienol.

As demonstrated in Table 1 and Figure 1, the chemical composition of FEO oil, based on the percentage composition, indicates the presence of both major and minor constituents. The major constituents include Alpha-phellandrene, which makes up 10.52% of the oil, followed by limonene/monoterpene at 7.31%. Other significant constituents include (1S,2S,3R,5S)-(+)-Pinanediol (3.41%), Thujone (3.25%), and 2,4-Cycloheptadien-1-one, 2,6,6-trimethyl- (2.91%), contributing to the oil aromatic complexity and potential therapeutic benefits. The minor constituents, each with a peak area of less than 3%, include various oxygenated monoterpenes and phenolic compounds, which add to the oil overall bioactivity. These minor components, despite their lower abundance, may play crucial roles in synergistic effects that enhance the oil pharmacological properties. Overall, the oil is predominantly composed of monoterpenes, with *alpha-phellandrene* and *limonene* being the most abundant constituents.

### 2.2. Percentage Wound Contraction

**Figure 2:**
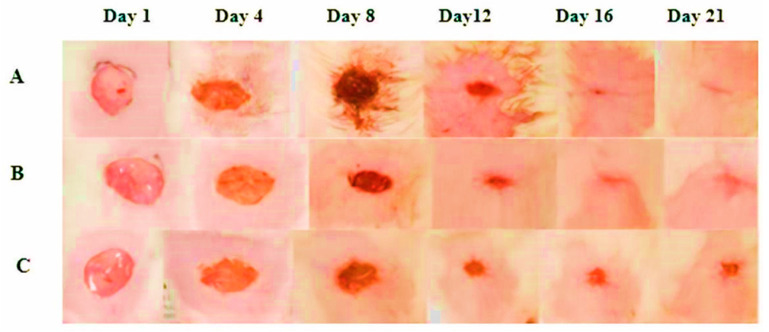
Representative images of wound healing across groups. The FEO (**A**), reference (**B**), and control (**C**) exhibited progressive wound healing, as illustrated by representative images captured from day 1 to day 21. Notably, the treatment group demonstrated accelerated wound closure, achieving full epithelialization by the end of the observation period.

All three groups excision wounds showed a progressive reduction in the area upon evaluation. However, the drug-treated group demonstrated 98.59 ± 0.2709% healing on the 16th day after the wound, compared to 95.13 ± 0.7579% for the reference-treated group and 91.36 ± 1.203% for the control group. The drug-treated group showed highly significant outcomes compared to the reference and control groups (Table 2, Figure 2).

### 2.3. Body Weight

Table 3 demonstrates that complete wound healing was observed in the FEO-treated group, accompanied by a notable increase in body weight (224.5 ± 3.274 g) and feed intake (12.46 ± 0.568 g) compared to the reference (204.7 ± 1.856 g, 10.79 ± 0.4017 g) and control groups (202.2 ± 4.110 g, 9.138 ± 0.2590 g).

### 2.4. Effect of FEO on Inflammatory Markers and Wound Healing Dynamics

The findings in Table 4 indicate that on day 21, inflammatory markers IL-1β and TNF-α were significantly reduced in the FEO-treated group compared to the standard drug and control groups. This decrease suggests that FEO effectively modulates the inflammatory response, contributing to improved wound healing. Table 4 further supports these results, highlighting the anti-inflammatory potential of FEO in comparison to the other treatments.

### 2.5. Effect of FEO on CD68 Level in FEO Treated Rats

CD68 was a glycoprotein and an indicator of wound-healing macrophages. The results of CD68 expression in the wound tissues of all experimental groups are illustrated in Table 4. The untreated control group exhibited a pronounced increase in CD68 levels compared to both the standard drug-treated and FEO-treated groups (Table 4). Notably, the reduction in CD68 levels was significantly more pronounced in the FEO-treated group than in the standard drug group. These findings highlight the superior efficacy of FEO in reducing CD68 levels, suggesting its potent anti-inflammatory properties and its pivotal role in accelerating wound healing.

### 2.6. Effect on Oxidative Stress Markers and Antioxidant Profile

**Figure 3:**
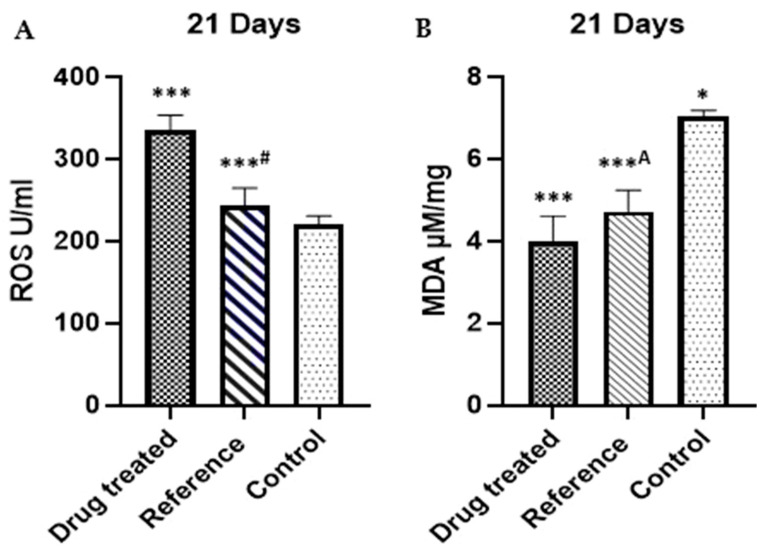

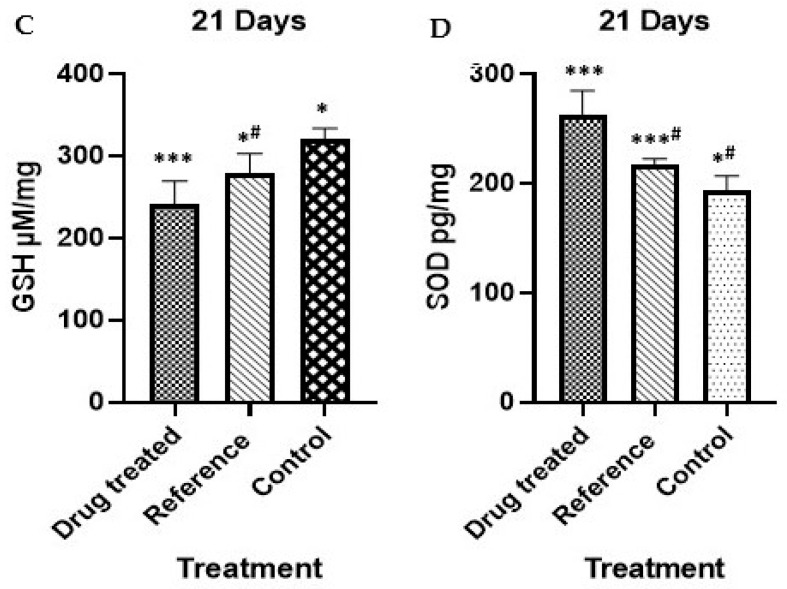
Effect of FEO on (**A**) ROS, (**B**) MDA, (**C**) GSH, and (**D**) SOD against reference and control group. *n* = 6; values are expressed as Mean ± SEM; *** *p* < 0.005 —FEO vs. control group, ***^#^ *p* < 0.05 —FEO vs. reference group, * *p* < 0.05 —FEO vs. reference group *^#^ *p* < 0.05 —reference vs. control, ***^A^ *p* < 0.005—Reference vs. control group. Data were analyzed by one-way ANOVA followed by Tukey’s multiple comparison test. Values of *p* < 0.05 are considered significant.

By day 21 post-wounding, the drug-treated group exhibited a significant increase in ROS levels (Figure 3A) compared to the reference and control groups. MDA levels (Figure 3B), a marker of lipid peroxidation, were lower in the drug-treated group, suggesting reduced oxidative stress. GSH levels (Figure Figure 3C) were lower in the drug-treated group, indicating an adaptive antioxidant response. Meanwhile, SOD activity (Figure Figure 3D) was significantly increased in the drug-treated group compared to the reference and control groups, highlighting its role in counteracting oxidative stress and promoting wound healing. These findings suggest that the drug treatment modulates oxidative stress by balancing ROS, MDA, GSH, and SOD levels, contributing to an improved healing process.

### 2.7. Histological Results

#### Measurement of Histopathological Changes

**Figure 4:**
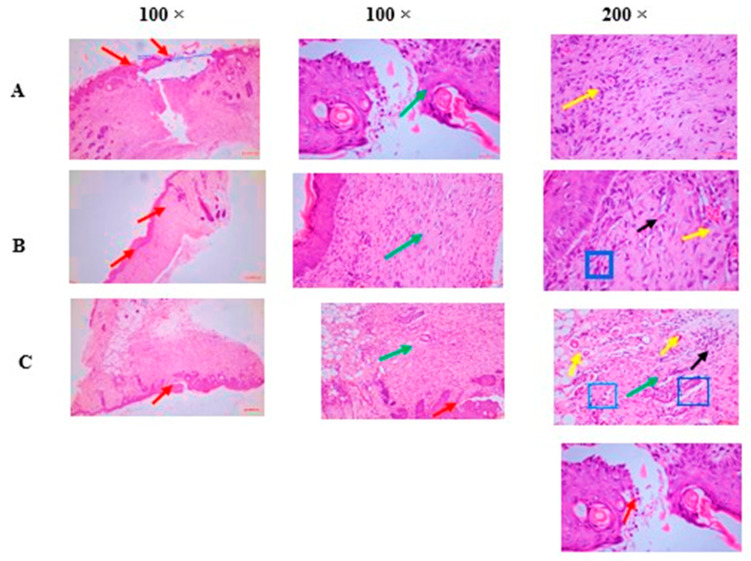
(**A**) FEO treated—partial re-epithelization of wound region with epidermal hyperplasia [red arrow] and moderate inflammatory response with granulation tissue proliferation in the dermal region surrounding the wound [green arrow] and mild angiogenesis or neovascularization [1–5 blood vessels one HPF]—[yellow arrow] was observed. The wound region surrounding the epidermal region was replaced by thick granulation tissue composed of a network of small blood vessels (yellow arrow—capillaries), fibroblasts [blue square] produce collagen and other extracellular matrix components), and various types of inflammatory cells [black arrow] predominantly plasma cells/lymphocytes followed by neutrophils. (**B**) Reference—complete re-epithelization of wound region with epidermal hyperplasia [red arrow] and moderate inflammatory response with granulation tissue proliferation in the dermal region surrounding the wound [green arrow] and mild angiogenesis or neovascularization [less than five blood vessels one HPF]—[yellow arrow] was observed. The wound region surrounding the epidermal region was replaced by thick granulation tissue composed of network of small blood vessels [yellow arrow—capillaries], fibroblasts (blue square] produce collagen and other extracellular matrix components), and various types of inflammatory cells [black arrow] predominantly plasma cells/lymphocytes followed by neutrophils. (**C**) Control—partial healing of wound in which partial re-epithelization of wound region with excessive accumulation of inflammatory exudates with necrotic materials in epidermal layers [red arrow] and mild inflammatory response with granulation tissue proliferation in the dermal region surrounding the wound [green arrow] and moderate angiogenesis or neovascularization [5 to 10 blood vessels one HPF]—[yellow arrow] was observed. The wound region surrounding the epidermal region was replaced by granulation tissue is composed of a network of small blood vessels [yellow arrow—capillaries], fibroblasts [blue square] produce collagen and other extracellular matrix components), and various types of inflammatory cells [black arrow] predominantly neutrophils followed by plasma cells/lymphocytes.

The histopathological changes were assessed using an arbitrary scoring system. A composite score was generated based on several key factors, with higher scores indicating poorer healing and lower scores reflecting better recovery. Scoring was observed in the following areas: inflammatory response, granulation tissue formation, re-epithelialization, angiogenesis, and collagen deposition, with confirmation through Masson’s trichrome (MT) staining. The histopathological changes for each group were calculated by averaging the individual scores across all time points (Table 5, Figure 4).

### 2.8. Immunohistochemical Staining

**Figure 5:**
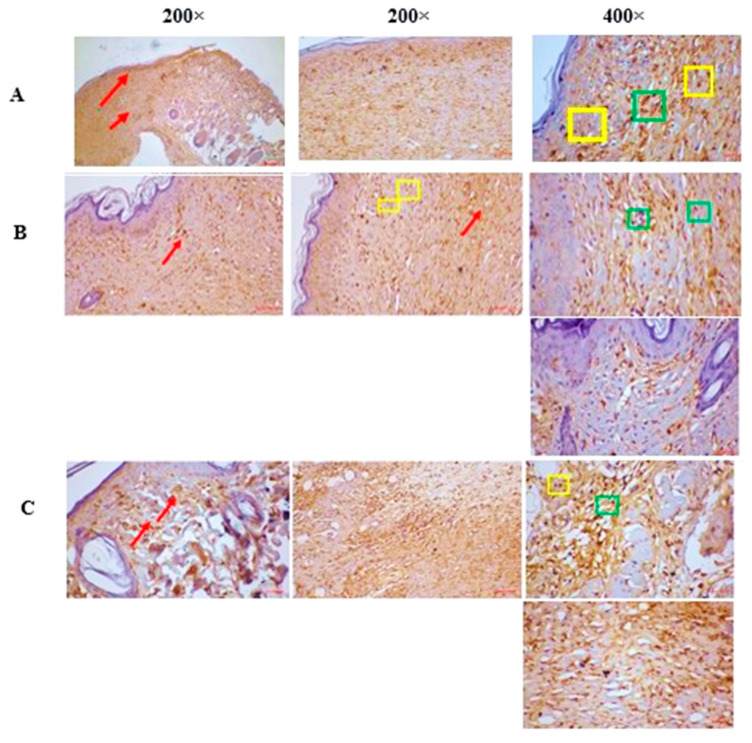
(**A**) FEO treated—moderate expression of caspase-3 in the partially healed wound region of epidermis/dermis region—red arrow. Moderate nucleo cytoplasmic expression of caspase-3 in the granulation tissue in the wound region especially fibroblast [green square] with immune cells [neutrophils/plasma cells/lymphocytes—yellow square] in the epidermal layers and dermal layer of skin. (**B**) Reference—moderate expression of caspase-3 in the wholly healed wound region of epidermis/dermis region—red arrow. Moderate nucleo cytoplasmic expression of caspase-3 in the granulation tissue in the wound region especially fibroblast [green square] with immune cells [neutrophils/plasma cells/lymphocytes—yellow square] in the epidermal layers and dermal layer of skin. (**C**) Control—mild expression of caspase-3 in the wholly healed wound region of epidermis/dermis region—red arrow. Mild nucleo cytoplasmic expression of caspase-3 in the granulation tissue in the wound region, especially fibroblast [green square] with immune cells [neutrophils/plasma cells/lymphocytes—yellow square] in the epidermal layers and dermal layer of skin.

The immunohistochemical analysis of caspase-3 expression across the three groups reveals distinct patterns associated with wound healing (Figure 5). In the FEO-treated group, moderate caspase-3 expression is observed in the partially healed wound, particularly in fibroblasts and immune cells within the granulation tissue. The reference group shows a similar level of caspase-3 expression but with more advanced wound healing, indicated by complete re-epithelialization. In contrast, the control group exhibits mild caspase-3 expression, with limited fibroblast involvement and reduced immune cell activity, corresponding with a more complete healing state. These findings suggest that FEO treatment modulates caspase-3 expression to potentially support wound healing through controlled apoptotic activity.

## 3. Discussion

The chemical characterization (Table 1 and Figure 1) of FEO using GC-MS analysis revealed the presence of both major and minor constituents, with alpha-phellandrene (10.52%) and limonene (7.31%) identified as the predominant compounds. Other notable constituents include (1S,2S,3R,5S)-(+)-Pinanediol (3.41%), thujone (3.25%), and 2,4-cycloheptadien-1-one (2.91%), contributing to the oil’s aromatic and therapeutic properties. These findings align with existing literature but exhibit variations in the relative abundances of specific components. For instance, a comparative study of commercial *Boswellia carteri* oils found alpha-pinene and limonene as major constituents, with alpha-phellandrene reported in lower concentrations [15]. Such differences may result from factors like geographical origin, extraction method, and resin harvesting practices [16]. These compositional variations are noteworthy, as even minor constituents can significantly influence the oil pharmacological properties through synergistic interactions. The dominance of monoterpenes in the FEO sample supports its observed anti-inflammatory and antioxidant activities, highlighting its potential utility in therapeutic applications.

The observed wound healing benefits of FEO in this study can be attributed to its bioactive components, such as *Boswellia* acids and various terpenoids, known for their potent anti-inflammatory and tissue-regenerative properties [17]. These compounds mitigate systemic inflammation, which is critical for optimizing nutrient absorption and utilization, thereby supporting tissue repair and recovery [18]. As demonstrated in Figure 2, the progressive wound healing observed with FEO treatment may be closely associated with these anti-inflammatory effects. Additionally, the enhanced feed intake and increased body weight, as indicated in Table 3, suggest that the active constituents of FEO positively influence metabolic processes essential for efficient tissue regeneration and overall recovery [19]. This systemic improvement may further contribute to the sustained wound healing outcomes observed in the treated group.

The ability of FEO to enhance wound closure, a fundamental marker of wound healing, can also be attributed to these active constituents. In this study, FEO application significantly accelerated wound closure and resulted in higher closure percentages compared to the control group, as illustrated in Table 2. This outcome aligns with previous findings demonstrating that boswellic acids and terpenoids stimulate key cellular processes such as fibroblast proliferation, keratinocyte migration, and collagen synthesis, essential mechanisms for effective tissue regeneration [20]. The histopathological analysis further supports this observation, revealing improved granulation tissue formation and dermal restructuring in the FEO-treated group. These effects are likely mediated by the synergistic actions of terpenoids, which enhance fibroblast activity and extracellular matrix deposition, ultimately contributing to more efficient wound healing [21].

Oxidative stress is another critical factor influencing wound healing. Excessive reactive oxygen species (ROS) and lipid peroxidation products, such as malondialdehyde (MDA), can impair cellular functions and delay tissue repair [22,23]. FEO exhibits strong antioxidant properties, reducing oxidative damage at wound sites, as demonstrated in Figure 3A–D. This effect is primarily attributed to its bioactive constituents, including *Boswellia* acids, terpenoids, alpha-thujone, isocaucalol, and hydroxylinalool, which scavenge ROS and inhibit lipid peroxidation [24]. By mitigating oxidative damage, FEO preserves essential wound-healing cells, such as fibroblasts and endothelial cells, ensuring more effective tissue regeneration. Although (-)-cis-sabinol and trans-verbenol lack direct evidence of caspase-3 inhibition, their general antioxidant properties may still contribute to the reduction in oxidative stress [25].

The regulation of inflammatory cytokines, particularly IL-1β and TNF-α, plays a critical role in orchestrating the wound healing process. While these cytokines are essential during the early inflammatory phase, their prolonged elevation can delay tissue repair and contribute to chronic inflammation [26]. FEO anti-inflammatory effects, as evidenced by the reduced levels of IL-1β and TNF-α at the wound site (Table 4), suggest the involvement of key terpenoids such as limonene, estragole, and gamma-terpinene. These compounds have been reported to suppress cytokine production through NF-κB inhibition, thereby preventing excessive inflammatory responses [27]. Additionally, terpinen-4-ol and carvacrol modulate macrophage activity, further supporting the resolution of inflammation [28]. Hydroxylinalool, a monoterpenoid component of FEO, has been found to exert antioxidant effects, protecting tissues from oxidative damage and aiding in the transition from the inflammatory to the proliferative phase of wound healing [29]. By modulating inflammatory cytokines, FEO creates a conducive microenvironment for tissue repair, ultimately promoting efficient wound closure and restoration of skin integrity [30,31].

Macrophages play a pivotal role in the wound healing process, particularly during the inflammatory and proliferative phases, by releasing cytokines like IL-1β and TNF-α to coordinate tissue repair. CD68 serves as a key marker for macrophage activation, indicating active immune response and tissue remodeling [32]. In this study, FEO modulated CD68 expression, as seen in Table 4, which reflects its role in regulating the inflammatory phase and facilitating a smooth transition to the proliferative stage. The oil constituents, including limonene, alpha-phellandrene, and gamma-terpinene, have been reported to enhance macrophage infiltration and phagocytic activity through CD68 upregulation [33]. Furthermore, terpinen-4-ol supports this process by promoting the release of pro-phagocytic factors, while germacrene D, known for its antimicrobial properties, aids macrophage-driven tissue clearance [34]. By modulating both cytokine activity and macrophage function, FEO supports efficient wound healing through enhanced immune response and tissue remodeling.

Apoptosis, a crucial mechanism for removing damaged or dysfunctional cells during wound healing, is tightly regulated by caspase-3 [35]. However, excessive caspase-3 activation during the inflammatory phase can result in the premature depletion of critical cells like keratinocytes, fibroblasts, and endothelial cells, consequently delaying tissue regeneration. FEO appears to modulate apoptosis by downregulating caspase-3 expression, thereby preserving these essential cells and ensuring a smooth transition into the proliferative phase. This downregulatory effect may be linked to components such as alpha-thujone, which has been reported to inhibit caspase-3 cleavage and prevent premature apoptosis [36]. Additional contributors to this apoptotic modulation include isocaucalol and hydroxylinalool, although their precise mechanisms remain less defined. The anti-inflammatory and antioxidant activities of (-)-cis-sabinol and trans-verbenol may further support wound healing by reducing oxidative stress and inflammation [37]. The observed anti-inflammatory response (Table 4) and antioxidant activity (Figure 3A–D) align with the downregulation of caspase-3 seen in Figure 5A–C. This controlled apoptotic regulation not only prevents excessive cell loss but also facilitates extracellular matrix deposition, wound contraction, and angiogenesis, promoting more efficient tissue repair [27].

Additionally, previous research indicates that essential oils often exhibit dose-dependent apoptotic effects, wherein low-to-moderate concentrations can inhibit apoptosis, while higher doses may induce it. For instance, studies reported that *Artemisia arborescens* oil significantly increased caspase-3 activation at high concentrations but had minimal effects at lower doses [38]. Similarly, *Linum usitatissimum* seed oil enhanced caspase-3 expression when applied at 250 μg/mL compared to 125 μg/mL [39]. Alpha-thujone, a major component of FEO, has demonstrated caspase-3 inhibition at lower concentrations, potentially aiding wound healing by preserving keratinocyte populations [40]. Future studies may explore these concentration-dependent effects to provide more clarity regarding the apoptotic modulation observed in this study.

Re-epithelialization, the process by which new epithelial cells cover the wound bed, represents a critical phase of wound healing [19]. The histopathological findings (Table 5, Figure 4A–C) indicate that FEO significantly accelerated re-epithelialization compared to the reference and control groups. This effect may stem from the oil anti-inflammatory and antioxidant properties, which help establish a favorable microenvironment for keratinocyte proliferation and differentiation. This observation aligns with findings from prior research, where essential oils with similar phytochemical profiles, such as monoterpenes and oxygenated terpenes, were shown to promote fibroblast migration, enhance extracellular matrix deposition, and reduce oxidative stress. The antioxidant activity of FEO may also facilitate tissue homeostasis by modulating ROS levels, which, when maintained within optimal ranges, serve as essential signaling molecules for keratinocyte migration and angiogenesis. The accelerated re-epithelialization observed in this study aligns with findings from previous investigations into essential oils with comparable phytochemical compositions.

## 4. Materials and Methods

### 4.1. Albino Rats

The central animal house provided adult male albino rats weighing 150–170 g. These rats were acclimated to a specific environment with a temperature of 25 ± 2 °C and a humidity of 55%. Rats were continuously assessed using 12 h cycles of light and dark. Rats were kept in separate housing and had unlimited access to tap water and standard laboratory food. Every animal procedure complies with the Experimental Animal Ethics Committee recommendations [41].

### 4.2. Experimental Design

For the wound healing study, three groups of healthy adult albino rats (*n* = 6) were used.

Group I—the control group received yellow soft paraffin
Group II—animals treated with standard (1% *w*/*w* silver sulfadiazine)
Group III—animals treated with test drug (10% *w*/*w* FEO)

### 4.3. Wounding (Morton and Malone Method)

Three sets of albino rats were created. Before wounding, the animals were given a 12-h fast and intraperitoneal anesthesia with a ketamine (50 mg/kg) and xylazine (10 mg/kg) combination. Rats were put on a surgical table in a prone position. After that, an electric shaver was used to remove the hairs on the back between the shoulders. After that, 70% ethanol was used to clean the region to preserve aseptic conditions and generate an excision wound. Using the Morton and Malone technique (1972), a circular incision with a diameter of around 2 cm (200 mm^2^) was performed in the dorsal thoracic area of rats in a semi-aseptic environment. Then, a full-thickness specimen made up of epiderm, derm, and hypodermic was removed from the skin using a scalpel. Sterile gauze was used to mop up any excess blood. All of the wounds were left exposed, and they were not bandaged during the research. Animals who displayed symptoms of illness were isolated and removed from the trial after being thoroughly monitored for any infections. Each animal was kept in its own home [42].

### 4.4. Source of Oil

The oil is sourced from the supplier, Naturalis Essential Oil Company, located in Manyata tech park, Karnataka-India. It is Manufactured and packaged (Mfg.Date: Aug-2024, Batch No: BX824) by TSBT International in Malur, India. As per manufacturer site information, the oil is extracted using a steam distillation process from the gums of the frankincense tree, ensuring a high-quality product. The oil is 100% natural, without any blending, dilution, or formulation, and is free from additives and other technical adjuvants.

### 4.5. Identification of Compounds in FEO by GCMS

The chemical composition of FEO was analyzed using gas chromatography-mass spectrometry (GC-MS) on a Shimadzu GCMS-QP2010 Plus system (Shimadzu Corporation, Kyoto, Japan) equipped with an HP-5MS capillary column (30 m × 0.25 mm i.d. × 0.25 µm film thickness, Agilent Technologies, Santa Clara, CA, USA. Prior to injection, the sample was diluted in hexane (1:10 *v*/*v*) and filtered through a 0.22 µm membrane filter. A 1.0 µL aliquot was injected in split mode (split ratio 40:1) at an injector temperature of 250 °C. Helium was used as the carrier gas at a constant flow rate of 1.0 mL/min. The oven temperature program started at 50 °C, held for 1 min, followed by a ramp of 8 °C/min until reaching 280 °C, where it was maintained for 2 min. The ion source operated at 200 °C with electron impact ionization (EI) at 70 eV, and mass scanning was performed in the range of 40–900 *m*/*z*. The total analysis time was 31 min. The identification of chemical constituents was carried out by comparing mass spectra with the NIST Library Version 8.0 (National Institute of Standards and Technology, Gaithersburg, MA, USA).

### 4.6. Preparation of Ointment

Ointments were prepared by incorporating 10% *w*/*w* of FEO in yellow soft paraffin obtained from a pharmacy. Levigation ensured a smooth and consistent texture by preparing the ointment on a slab. Silver sulfadiazine ointment (1% *w*/*w*) was used as the standard control, while the yellow soft paraffin was the negative control.

### 4.7. Treatment

The test and usual medication were administered daily with a sterile spatula until the epithelium was completely covered. Every day, this was carried out cautiously to prevent dosage variations. The surfaces of each wound were carefully and methodically cleaned with a tampon soaked in physiological serum before administering the therapies. A graph paper measured the reported wound areas on days 1, 4, 8, 12, 16, and 21. On day 21, the healed wound and the surrounding skin were removed, and they were then divided into two equal sections for histopathological study and the antioxidants measurement procedure.

### 4.8. Evaluation Parameters

*Percentage wound contraction:* Measurement of wound contraction was used for evaluation. The wound was traced every four days (days 1, 4, 8, 12, 16, and 21) to determine the wound closure rate using transparency paper and a permanent marker. Each tracing area (mm^2^) inside its borders was calculated planimetrically. All of the wounds were digitally photographed at the same intervals. ImageJ (version 0.5.5) tools were used to measure the area of the wound [43].

% wound contraction = (1 wound area on day zero − wound area on particular day/wound area on day zero) × 100 [43].

### 4.9. Biochemical Evaluation

#### 4.9.1. Sample Collection

Blood samples from the rats in each group were collected, and the EDTA-free tube orbital sinus method was used to separate the sera for assays of CD68, IL1β, and tumor necrosis factor-alpha (TNF-α) [44].

#### 4.9.2. Determination of the Rat Serum TNF-α and IL-1β Levels

As instructed by the manufacturer, a commercial kit for an enzyme-linked immune sorbent assay (ELISA) was used to quantify the amounts of TNF-α and IL-1β in serum rats. A standard curve was used to measure the cytokine concentrations, which were then reported as Pg/mL [43,45].

#### 4.9.3. Estimation of CD68 in the Rat Serum

Following the manufacturer’s instructions, a commercial enzyme-linked immune sorbent assay (ELISA) kit was used to measure the CD68 level in serum rats. Using a standard curve, the amount of macrophage CD68 was determined and reported as ng/mL [46].

#### 4.9.4. Antioxidant Activity

A glass homogenizer was used to homogenize skin specimens in 1 mL saline/g tissue. Before analysis, the supernatant was kept at −80 °C after the homogenates were centrifuged at 10,000× *g* for 30 minutes at 4 °C. ELISA kits (Andy Hua Tai, Beijing, China) were used to measure the levels of ROS, MDA, GSH, and SOD protein (antioxidant enzymes) by the manufacturer instructions [43,45].

### 4.10. Histopathological Evaluation

#### 4.10.1. Sample Collection

Dorsal skin samples were obtained from each animal, fixed in buffered formalin, processed with xylene and alcohol, and subsequently embedded in paraffin blocks. To determine the density of collagen fibers, tissue slices of 4 µm in thickness were uniformly stained with hematoxylin/eosin and a particular Masson’s trichrome. Mounted slides were analyzed and photographed utilizing the Leica Application Suite (Leica Microsystems, Wetzlar, Germany, a light microscope) [47].

#### 4.10.2. Immunohistochemical Staining

Cell apoptosis in wound tissue was detected by DAB staining, hematoxylin counterstaining, and caspase-3 immunohistochemical labeling using anti-caspase-3 antibody [EPR18297] (Abcam) (Santa Cruz Biotechnology, Dallas, TX, USA). A brownish-yellow hue was seen in the positive cells. Five locations of interest located inside wound tissues in three sections per animal were used to measure positive cells. Software called Image-Pro Plus 6.0 was used to make the measurements. By normalizing the total caspase-3+ to the total number of cells, the percentage of caspase-3+ cells was calculated [47].

### 4.11. Statistical Analysis

The means ± SEM were used to depict the data. Using the software application GraphPad Prism (version 8.00), the data were subjected to Tukey’s comparison test after a one-way analysis of variance (ANOVA). At *p* < 0.05, the significance threshold was taken into account.

## 5. Conclusions

FEO has demonstrated remarkable potential in promoting wound healing by effectively regulating key cellular and molecular mechanisms. Its ability to modulate apoptosis through the downregulation of caspase-3 expression facilitates a crucial transition from the inflammatory to the proliferative phase of wound healing. This regulatory action on caspase-3, possibly by its anti-inflammatory and antioxidant properties, minimizes prolonged inflammation, reduces oxidative stress, and creates an environment conducive to tissue regeneration. Furthermore, FEO enhances fibroblast proliferation, collagen synthesis, and angiogenesis, which are vital for wound contraction and repair. In addition, the anti-inflammatory and antioxidant properties also help mitigate excessive immune responses, ensuring a balanced healing process. Histological analysis confirmed improved granulation tissue formation, reduced inflammatory cell infiltration, and increased epithelialization in treated wounds. By targeting apoptosis and promoting cellular survival, FEO serves as a dual-action therapeutic agent in wound management. These findings highlight its significant role in accelerating the natural healing process and improving outcomes in tissue repair.

## Figures and Tables

**Table 1:** Compounds from the Frankincense oil GCMS data with a peak area percentage > 1.0%.

Retention Time (min)	Compound Name	Peak Area (%)	Molecular Formula	Chemical Class
5.578	1,3-cyclohexadiene, 2-methyl-5-(1-methylethyl)-	10.52	C_10_H_16_	Alpha-phellandrene
7.347	Cyclohexene, 1-methyl-4-(1-methylethenyl)-	7.31	C_10_H_16_	Limonene/Monoterpene
12.984	(1s,2s,3r,5s)-(+)-pinanediol	3.41	C_10_H_18_O_2_	Serine Protease
9.054	Thujone	3.25	C_10_H_16_O	Alpha-Thujone
12.863	2,4-cycloheptadien-1-one, 2,6,6-trimethyl-	2.91	C_10_H_14_O	Eucarvone/Monoterpenoid
10.645	Estragole	2.82	C_10_H_12_O	Estragole/Phenylpropanoid
6.96	1,4-cyclohexadiene, 1-methyl-4-(1-methylethyl)-	2.72	C_10_H_16_	Gamma-Terpinene
10.129	Bicyclo[3.1.0]hexan-3-ol, 4-methylene-1-(1-methylethyl)-, (1.alpha.,3.alpha.,5.alpha.)-	2.39	C_10_H_16_O	(-)-Cis-Sabinol
13.102	Sobrerol 8-acetate	1.89	C_12_H_20_O_3_	Monoterpenoids
14.079	(2e,4s,7e)-4-isopropyl-1,7-dimethylcyclodeca-2,7-dienol	1.89	C_15_H_26_O	Germacrene D
5.643	1,4-cyclohexadiene, 1-methyl-4-(1-methylethyl)-	1.85	C_10_H_16_	Gamma-Terpinene/Cyclohexadiene
10.956	3-buten-2-one, 4-(2,6,6-trimethyl-2-cyclohexen-1-yl)-	1.74	C_13_H_20_O	Alpha-Ionone/Methyl Ketone
9.143	Trans-verbenol	1.7	C_10_H_16_O	Trans-Verbenol
14.277	Isocaucalol	1.68	C_15_H_26_O_3_	Isocaucalol
10.256	3-cyclohexen-1-ol, 4-methyl-1-(1-methylethyl)-	1.5	C_10_H_18_O	Terpinen-4-Ol
12.622	Phenol, 2-methyl-5-(1-methylethyl)-	1.5	C_10_H_14_O	Phenol/Carvacrol
7.712	5-isopropyl-2-methylbicyclo[3.1.0]hex-3-en-2-ol	1.2	C_10_H_16_O	Monoterpenoid
11.584	(1s,2s,3r,5s)-(+)-pinanediol	1.13	C_10_H_18_O_2_	2,3-Pinanediol
13.9	2,7-octadiene-1,6-diol, 2,6-dimethyl-	1.01	C_10_H_18_O_2_	8-Hydroxylinalool

**Table 2:** Effect of FEO on % wound closure against reference and control group.

Group	% Wound Closure (Days)				
	Day 4	Day 8	Day 12	Day 16	Day 20
FEO	42.02 ± 2.112 **	57.21 ± 1.873 ***^#^	86.00 ± 1.689 ***^#^	98.59 ± 0.2709 ***	100 ± 0.002 ***
Reference	20.05 ± 3.129 ***^#^	44.32 ± 2.192 ***	87.90 ± 1.620 ***	95.13 ± 0.7579 *^#^	99.12 ± 0.192 **^#^
Control	26.69 ± 2.334	47.98 ± 2.135 *^a^	85.79 ± 0.9587	91.36 ± 1.203 *^a^	98.29 ± 0.226 **^a^

*n* = 6; group, ***^#^ *p* < 0.005—FEO vs. reference group, *** *p* < 0.005—FEO vs. control group, ** *p* < 0.05—FEO vs. control group, **^#^ *p* < 0.05—FEO vs. reference group, **^a^ *p* < 0.05—reference treatment vs. control group, *^#^ *p* < 0.05—FEO vs. reference group, *^a^ *p* < 0.05—reference vs. control group. Data were analyzed by one-way ANOVA followed by Tukey’s multiple comparison test. Values of *p* < 0.05 are considered significant.

**Table 3:** Effect of FEO on body weight and feed intake against reference and control group.

Parameters	Body Weight	Feed Intake
FEO	224.5 ± 3.274 ***	12.46 ± 0.568 ***
Reference	204.7 ± 1.856 **	10.79 ± 0.4017 *
Control	202.2 ± 4.110	9.138 ± 0.2590 *^#^

*n* = 6; values are expressed as mean ± SEM, *** *p* < 0.005—FEO treatment vs. control group, ** *p* < 0.05—FEO treated vs. reference group, * *p* < 0.05—FEO treated vs. reference group, *^#^ *p* < 0.05—reference vs. control group. Data were analyzed by one-way ANOVA followed by Tukey’s multiple comparison test. Values of *p* < 0.05 are considered significant.

**Table 4:** Effect of FEO on CD68, TNF-α and IL-1β against reference and control group.

Parameters	CD68 (ng/mL)	TNF-α (pg/mg)	IL-1β (pg/mg)
FEO	23.33 ± 1.054 ***	358.3 ± 20.07 ***	666.7 ± 44.10 **
Reference	26.83 ± 0.6009 **^#^	491.7 ± 37.45 *	858.3 ± 41.67 *
Control	31.83 ± 1.014 *^#^	650 ± 42.82 *^#^	983.3 ± 60.09

*n* = 6; values are expressed as mean ± SEM; *** *p* < 0.005— FEO vs. control, ** *p* < 0.005 —FEO vs. control group, **^#^ *p* < 0.05 —reference vs. control group, *^#^ *p* < 0.05— reference vs. control, * *p* < 0.005— FEO vs. reference group. Data were analyzed by one-way ANOVA followed by Tukey’s multiple comparison test. Values of *p* < 0.05 are considered significant.

**Table 5:** Effect of FEO on histopathological changes against reference and control group.

Histopathological Changes	Average Score		
	FEO	Reference	Control
Inflammatory response	1	1	1
Granulation tissue formation	2	1	1
Re-epithelization	1	2	1
Angiogenesis	1	2	3
Collagen deposition	1	1	1

*n* = 6; group, measurement of histopathological changes for inflammatory response, granulation tissue formation, re-epithelization, angiogenesis, and collagen deposition.

## Data Availability

The original contributions presented in this study are included in the article. Further inquiries can be directed to the corresponding author.
